# Quackery and Superstition

**Published:** 1873-04

**Authors:** 


### Editorial.

### Quackery and Superstition.

Not long since a Dr. (?) came to our city who advertised himself as Dr. John T. Teasdill, from Lockport. (What especial virtue there is in coming from Lockport we are unable to conceive.) The learned individual claimed to cure most of the diseases to which flesh is heir. Among other ailments which he professed to cure "with or without medicine, or by simply laying hands on the patient," was "reptiles and other animals" in the stomach. His almost invariable diagnosis was "a frog in the stomach," and he presented numerous letters from different persons testifying that he had at various times removed frogs, lizards, snakes, etc., etc., from their stomachs.

This erudite doctor of medicine was found dead in his rooms a few days since, and suspended from his neck were found the following charms: a mule's shoe, a frog's skin stuffed with cotton, an old English shilling, and the kernel of a filbert. Each article had several wrappings of cotton, and were, without doubt, *effective* in the *cure* of diseases.

It would seem strange at first glance that in the nineteenth century, which boasts so much of its civilization and scientific attainments, there could be found a man who would believe in these senseless charms; or men and women so devoid of common sense as to become his dupes. But when we think how the public are being duped every day by quacks who pretend to wonderful cures, and count their patients among the wealthy and apparently intelligent, it does not seem strange that this obsure old "Frog Doctor" should succeed in persuading a few unknown and illiterate individuals that there was a frog in their stomach, and that he had the power to remove it.

He is dead, yet there remain in our midst others like him, and, as far as law or custom controls the matter, they have an equal right with the most learned physician in the State to call themselves doctors.

